# Endometriosis and Infertility: How and When to Treat?

**DOI:** 10.3389/fsurg.2014.00024

**Published:** 2014-07-02

**Authors:** Anis Fadhlaoui, Jean Bouquet de la Jolinière, Anis Feki

**Affiliations:** ^1^Service de gynécologie obstétrique, HFR Fribourg – Hôpital Cantonal, Fribourg, Switzerland

**Keywords:** endometriosis, infertility, female, laparoscopic surgery, IVF, hormonal therapy

## Abstract

Endometriosis is defined as the presence of endometrial-like tissue (glands or stroma) outside the uterus, which induces a chronic inflammatory reaction. Although endometriosis impairs fertility, it does not usually completely prevent conception. The question of evidence based-medicine guidelines in endometriosis-associated infertility is weak in many situations. Therefore, we will highlight in this issue where the challenges are.

## Introduction

Ovulatory disorders, tubal obstruction, and semen abnormalities account in nearly 75% of infertile couples. The remaining 25% of infertility is due to endometriosis (in up to 40% of the cases) or classified as unexplained (Table 1) (1).

**Table 1:** **Frequency of diseases associated with infertility (1)**.

	Diseases	Percentage
Female partner	Ovulatory disorders	25–27
	Endometriosis	5–15
	Pelvic adhesions	12
	Tubal occlusion	11
	Other tubal abnormalities	11
	Hyperprolactinemia	7
Male partner	Abnormal semen analysis	25
Unexplained		17
Other		4

Endometriosis is defined as the presence of endometrial-like tissue (glands or stroma) outside the uterus, which induces a chronic inflammatory reaction (2). The exact prevalence of endometriosis is unknown but estimated to range from 2 to 10% in women of childbearing age. Its prevalence rises up to 50% in women with infertility (3).

In women with endometriosis, there is a reduced monthly fecundity rate (2–10%) compared with fertile couples (15–20%) (4). Although endometriosis impairs fertility, it does not usually completely prevent conception. The question of evidence based-medicine guidelines in endometriosis-associated infertility is weak in many situations. Therefore, we will highlight in this issue where the challenges are.

## Endometriosis and Reproduction

The fecundity in the control groups of women with endometriosis attempting to become pregnant naturally was approximately half that of a group with pure unexplained infertility without endometriosis (5). A large multicentric prospective study (6) showed a reduced fecundity in women with minimal endometriosis. Although there is a substantial evidence for relationship between endometriosis and infertility, a causal relationship has not been established. The mechanisms for endometriosis-related infertility are not fully understood and seem to be different in different stages of endometriosis.

### Mild or minimal endometriosis and infertility

The mechanisms underlying reproductive failure are subtle and remain controversial, especially in cases where ovaries and fallopian tubes are normal. However, the following effects on reproduction could be noticed:

- A toxic effect on gametes, embryos, and impairment of tubal motility: endometriotic implants secrete pro-inflammatory cytokines (IL-1β, IL-8, IL-6, and TNF α), estradiol, and progesterone which attract macrophages, vascular endothelial growth factor (VEGF), and interleukin-8, thus creating an inflammatory state impairing fertility (7, 8).
- An abnormal follicular environment, high in cytokines (9).
- Increased rate of apoptosis in granulosa cells (10, 11).
- An enhanced ability to phagocytose sperm by peritoneal macrophages (12).
- A reduced rate of fertilization in women undergoing ART (13–17).
- An impairment of implantation rates and endometrial receptivity owing to the local inflammatory state and to an excessive production of antibodies to endometrial antigens (18).

### Moderate to severe disease and reproduction

In addition to above mentioned factors there is (19):

- An impairment of oocyte release owing to pelvic adhesions and endometriomas.
- An impairment of tubal transport.
- A blockage of sperm migration.

## Medical versus Surgical Treatment: What is the Best Choice and What Comes First?

The current debate is about whether it is necessary to medically treat, operate, and/or combine both treatments in infertile women.

### Medical treatment indication in reproductive medicine

In case of spontaneous conception, Hughes et al. (20) showed, through a large meta-analysis that ovarian suppression [oral contraceptive pill (OCP), GnRH agonists, Medroxyprogesterone acetate, Danazol] is not recommended for women with endometriosis and wishing to conceive, since there is no difference in spontaneous pregnancy or live births rates when compared to placebo or no treatment.

Since surgery may not remove microscopic disease, hormonal treatments have been used to suppress disease and to prevent recurrence. A meta-analysis comparing surgery plus hormonal treatment (GnRH agonists, Danazol, Medroxyprogesterone acetate) versus surgery plus placebo or no treatment showed no difference in pregnancy rates.

In infertile women with endometriosis, the Guideline Development Group (GDG) recommendation to clinicians is not to prescribe adjunctive hormonal treatment before surgery to improve spontaneous pregnancy rates, as suitable evidence is lacking (19). It is important to realize that clinicians should not withhold hormonal treatment for symptomatic women in the waiting period before undergoing surgery or medical assisted reproduction (19).

### Surgical treatment

Surgery’s aim is to remove macroscopic endometriosis implants and restore normal pelvic anatomy. However, surgery may not be able to completely restore pelvic anatomy or to stop inflammatory process. Hence, it is important to weigh up benefits versus harm of surgical procedure. Laparoscopy is preferred to laparotomy because of advantages of minimal tissue damage, of magnification, of faster recovery, and shorter hospital stay (21).

#### Is there a benefit of surgical treatment of stage I–II of endometriosis and successful pregnancy rate?

Several studies demonstrated that, in infertile women with endometriosis stage I/II of the American Fertility Society/American Society for Reproductive Medicine (AFS/ASRM), clinicians should perform operative laparoscopy (excision or ablation of endometriosis lesions) including adhesiolysis, rather than performing diagnostic laparoscopy only, since there is a positive effect in regards to live birth and ongoing pregnancy at 20 weeks of amenorrhea (OR 1.64; 95% CI 1.05–2.57) (19, 22).

According to ESHRE guidelines, and concerning management of women with stage I–II of endometriosis, clinicians may consider CO_2_ laser vaporization of endometriosis, instead of monopolar electro-coagulation, since laser vaporization is associated with higher cumulative spontaneous pregnancy rates (23).

#### Is there a benefit of surgical treatment of stage III–IV of endometriosis and successful pregnancy rate?

There is no randomized controlled trial or meta-analysis to assess whether surgery is positively effective or not on pregnancy rates in moderate to severe endometriosis. The lack of randomized trials or meta-analysis is not due to lack of research effort but to the unethical aspect of such studies that is to do nothing to a patient with stage III or IV endometriosis who is already under anesthesia could be ethically unacceptable.

There are many non-randomized uncontrolled studies with results varying from a postoperative pregnancy rate of 30–67% (24). Three high quality prospective cohort-studies (19, 25) showed crude spontaneous pregnancy rates of 57–69% (moderate endometriosis) and 52–68% (severe endometriosis) after laparoscopic surgery, which are much higher than the crude spontaneous pregnancy rates of 33% (moderate) and 0% (severe) after expectant management reported in a study by Vercellini et al. (19).

There is conflicting evidence to determine whether removal of recto-vaginal lesions improves spontaneous pregnancy rates (2). Moreover, such a kind of aggressive surgery is accompanied by a high rate of complications (26, 27).The discrepancy in results between the different stages of the disease shows no correlation between the AFS classification and the outcomes in terms of fertility. Thus it is necessary to define a phenotypic profile of the lesions (28). The major benefit of surgery is achieved shortly after the first attempt because severe peri-ovarian adhesions will generally recur and will limit tubal pick-up of the ovum. If initial surgery does not result in pregnancy, subsequent surgical procedures are not likely to be effective for increasing fecundability.

A systematic review demonstrated a halving of pregnancy rates after re-operative surgery compared with first line surgery (22% for repetitive surgery versus 40% after primary surgery) (29). The decision for re-operative surgery versus IVF must be made on symptoms, the presence of complex cysts requiring histological diagnosis, age, ovarian reserve, male factor infertility, and availability of skilled surgeons (24).

### How should we behave with ovarian endometrioma in case of infertility?

According to the ESHRE Guideline (19) in infertile women with ovarian endometrioma of >3 cm in size, surgeons should perform excision of endometrioma capsule instead of ablative surgery that is drainage and electro-coagulation of the endometrioma wall since it increases the spontaneous postoperative pregnancy rate.

Excision of endometriomas involves the opening of the cyst (using scissors or electrosurgical or laser energy). After identifying the plane of cleavage between the cyst wall and ovarian tissue, the cyst wall is then excised or “stripped away” by applying opposite bimanual traction and counter actin with two grasping forceps. The ovarian edges could be sutured or inverted by light application of bipolar coagulation or kept as they are. Ablative surgery also involves the opening and drainage or fenestration (making a window in the wall of the cyst) of the endometrioma, followed by the destruction of the cyst wall using either electrosurgical current, cutting or coagulating current or a form of laser energy.

A study by Donnez et al. (30) showed that a combined technique of excisional (cystectomy) and Laser ablative surgery without ovarian suture could be the best compromise for sparing ovarian reserve.

The ESHRE guideline (19) for the management of women with endometriosis, recommended that clinicians should counsel infertile women with endometrioma regarding the risks of reduced ovarian function after surgery and the possible loss of the ovary. The decision to proceed with surgery should be considered carefully if woman has had previous ovarian surgery.

### Is there any association between endometrioma and risk of ovarian cancer?

The ESHRE’s GDG concluded that there is no evidence that endometriosis causes cancer, though some cancers are slightly more common in women with endometriosis such as non-Hodgkin’s lymphoma and ovarian cancer (19). A very large study (31) showed a higher risk of histological subtypes of ovarian cancer in case of endometriosis. Self-reported endometriosis was associated with significantly increased risk of clear cell ovarian cancer (OR 3.05, 95% CI 2.43–3.84), low-grade serous ovarian cancer [OR 2.11, 95% CI 1.39–3.2 (*p* < 0.0001)], and endometrial invasive ovarian cancer [OR 2.04, 95% CI 1.67–2.48 (*p* < 0.0001)]. Clinicians should be aware of this increased risk and future efforts should be focused on understanding the mechanisms that might lead to malignant transformation of endometriosis so as to help identify subsets of women at increased risk of ovarian cancer.

### Does endometriosis impact ART outcomes?

**Figure 1:**
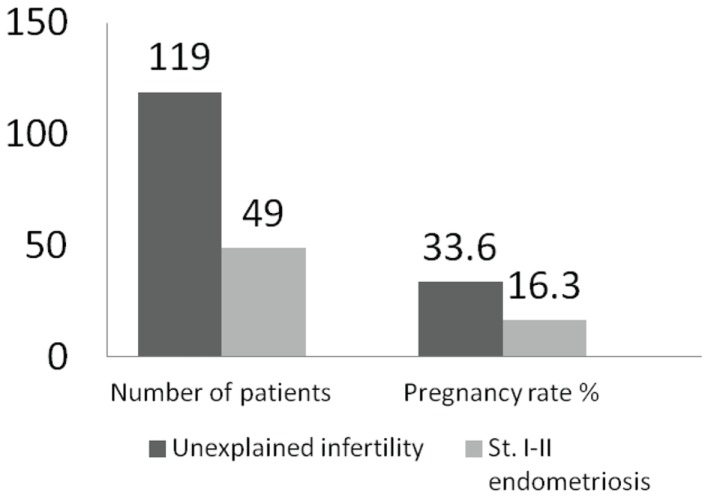
**Outcomes of COH–IUI in minimal or mild endometriosis (**33).

Minimal or mild endometriosis alters the outcome of controlled ovarian hyperstimulation (COH) in intra-uterine insemination (IUI) (32–38) with up to 30% reduction in pregnancy rate (Figure 1).

Two randomized controlled trials (24) supported the view that COH–IUI is better than no treatment for endometriosis. Tummon and co-workers found that cumulative live birth rate was fivefold higher following COH–IUI. In a systematic review and meta-analysis about endometriosis and IVF, Harb et al. (39) found:

- There is 7% reduction in fertilization rate in stage I/II endometriosis, and no difference in fertilization rate for stage III/IV endometriosis when compared to controls (IVF in women without endometriosis).
- There is no difference in implantation rate for stage I/II endometriosis when compared to controls, and 21% reduction in implantation rate for stage III/IV endometriosis.
- No difference in clinical pregnancies for stage I/II when compared to controls however there is 21% reduction in clinical pregnancies for stage III/IV endometriosis compared to controls.
- There is no statistical difference in live birth for all stage’s endometriosis.

Concerning ovarian endometriosis, since they are dealing with all stages of endometriosis, studies show different opinions, some found no impact (40–42), and others found a decreased response but no impact on IVF outcome (15, 16, 43–45) and finally some others found a decreased IVF outcome depending on the endometriosis severity (19, 46, 47).

Barnhart et al., in a meta-analysis of 22 observational studies, showed that women with endometriosis have poorer IVF outcomes (the number of oocytes collected and the number of fertilized oocytes) than women with tubal infertility (OR 0.56; 95% CI 0.4–0.7). Besides, women with more severe disease had worse outcomes than women with minimal-mild endometriosis (19).

More and more papers are reporting a reduction in ovarian reserve after laparoscopic surgery for endometriomas. Indeed, very frequently, normal ovarian tissue is excised with the endometrioma wall. Preservation of ovarian tissue (48), oocytes, or embryos cryopreservation (emergency IVF) (49) should be considered in all patients at serious risk of future fertility impairment as in case of cancers undergoing cytotoxic chemotherapies.

### Does medical treatment should be before or after surgery in patient with endometriosis undergoing ART?

In ART, the pre-treatment with GnRH agonists significantly increased live birth rate compared with no pre-treatment (OR 9.19; 95% CI 1.08–78.22) (50). The very wide confidence interval around the point estimate caused some doubt on the strength of the conclusions.

A study (51) suggested that ART outcomes following OCP pre-treatment in women with endometriosis are comparable with the outcomes of age-matched controls without endometriosis, thus showing a positive effect.

In one hand, in infertile women with stage I/II endometriosis, an IUI with gonadotropins controlled ovarian stimulation (COS) should be performed, instead of expectant management and instead of IUI alone as it increases respectively 5.6 and 5.1 times live birth rates. On the other hand, clinicians may consider performing IUI with COS within 6 months after surgical treatment, since pregnancy rates are similar to those achieved in unexplained in unexplained infertility (19).

However, it is less clear whether surgery for minimal – mild endometriosis prior to COH–IUI improves the success rate (24).

The influence of endometriosis on the success rate of IVF/ICSI in not unequivocal. Barnhart et al. found that pregnancy rates after IVF/ICSI were lower in patients with stage III/IV endometriosis as compared to those with tubal factor (19). However, some large databases (The society for assisted reproductive technology – SART – and the human fertilization and embryology authority) noted that endometriosis does not adversely affect pregnancy rates. It seems that GnRH antagonist protocol is not inferior to GnRH agonist protocol in women with stage I/II endometriosis and endometrioma (19). The GDG of the ESHRE recommends the use of ART for infertility associated with endometriosis, especially if tubal function is compromised or if there is male factor infertility and/or other treatments have failed (19).

In a Cochrane review, it appears that down-regulation for 3–6 months with GnRH agonists in women with endometriosis increases the odds of clinical pregnancy by more than fourfold (50).

The benefit of surgery for endometriomas prior to IVF is still uncertain (Table 2). A number of concerns have been raised as arguments for surgery. However, the available evidence appears to alleviate these concerns:

- There is no reduced ovarian responsiveness with COH in women or ovaries with endometriosis (24).
- There is no risk of growth or rupture of endometriomas with COH (24).
- To date, there are no studies that proved an increased risk of abscess formation following oocyte retrieval in women with endometriomas (24). Clinicians may use antibiotic prophylaxis at the time of oocyte retrieval, although the risk of abscess is low (19).
- Ovarian surgery seems to reduce the number of oocytes retrieved, to reduce the peak estradiol levels and to increase total FSH requirement. It has been reported that ovarian surgery can lead to ovarian failure in 13% of the cases (52, 53). Beside the later, in infertile women, resection of endometriomas larger than 3 cm does not seem to improve pregnancy rates (19, 54–56), thus the GDG according to ESHRE guidelines recommended to consider cystectomy prior to ART to improve endometriosis-associated pain or the accessibility of follicles 22. The decision to proceed with surgery should be considered carefully if women have had previous ovarian surgery.
- Concerning deep endometriosis, there is no evidence to recommend performing surgical excision of deep nodular lesions prior to ART, to improve reproductive outcomes. However, these women often suffer from pain, requesting surgical treatment (19, 55).
- ART treatments do not seem to increase the recurrence rate of endometriotic lesions or symptoms (19).

**Table 2:** **Risk and benefits of observational and surgical management of endometriomas (19)**.

Observational	Surgery
**BENEFITS**	
Avoid surgery	Exclude malignancy
Low FSH doses	Relieve symptoms
Increased E2	Reduce the risk of cyst complications
Increased follicles	Facilitate transvaginal access to ovarian follicles
**RISKS**	
Pain	Ovarian failure because of destruction of normal tissue
No histological diagnosis	Reduced number of egg collected
Pelvic infection following oocyte retrieval	Risks of surgery

## Conclusion

Endometriosis is a common disease in infertile women. It can affect fertility in many ways and at different levels. Medical treatment of endometriosis does not improve spontaneous pregnancy rates, whereas there is evidence that surgery is beneficial in minimal-mild endometriosis. There is controversial evidence regarding removal of endometriomas owing to the potential impact on ovarian reserve, but there are benefits of this surgery such as pain relief.

Other RCT are required to assess the potential effects of aggressive surgery and re-operative procedures. ART improves pregnancy rates as compared with no treatment, but the pregnancy rates remain lower than that of endometriosis-free women. Medical, surgical, and ART treatments do not need to occur separately and many women may benefit from a combination of these three approaches.

## Conflict of Interest Statement

The authors declare that the research was conducted in the absence of any commercial or financial relationships that could be construed as a potential conflict of interest.
